# Long-term cardiovascular outcome in women with preeclampsia in Korea: a large population-based cohort study and meta-analysis

**DOI:** 10.1038/s41598-024-57858-6

**Published:** 2024-03-29

**Authors:** Eun-Saem Choi, Young Mi Jung, Dayoung Kim, Su Eun Cho, Eun Sun Park, Chan-Wook Park, Joong Shin Park, Jong Kwan Jun, Seung Mi Lee

**Affiliations:** 1 Department of Obstetrics and Gynecology, Seoul National University Hospital, Seoul, Republic of Korea; 2 Department of Obstetrics and Gynecology, College of Medicine, Guro Hospital, Korea University, Seoul, Republic of Korea; 3 Department of Obstetrics and Gynecology, Seoul National University College of Medicine, Seoul, 03080 Republic of Korea; 4 Seoul National University College of Medicine, Seoul, 03080 Republic of Korea; 5 The Institute of Reproductive Medicine and Population, Medical Research Center, Seoul National University College of Medicine, Seoul, 03080 Republic of Korea; 6 Innovative Medical Technology Research Institute, Seoul National University Hospital, Seoul, Republic of Korea

**Keywords:** Cardiology, Diseases, Medical research, Risk factors

## Abstract

Recent studies reported the long-term cardiovascular risk of preeclampsia. However, only a few studies have investigated the association between preeclampsia and long-term cardiovascular disease in Asian populations, although there could be racial/ethnic differences in the risk of cardiovascular diseases. Therefore, we aimed to evaluate the long-term effects of preeclampsia on cardiovascular disease in an Asian population. This study included 68,658 parous women in the Health Examinees Study (HEXA) cohort of South Korea and compared the risk of long-term cardiovascular disease, including ischemic heart disease and stroke, according to the history of preeclampsia. We also performed a meta-analysis combining current study data with data from existing literature in the Asian population. Among the study population, 3413 (5.23%) women had a history of preeclampsia, and 767 (1.12%) and 404 (0.59%) women developed ischemic heart disease and stroke for 22 years. Women with a history of preeclampsia were at a higher risk for both ischemic heart disease (adjusted hazard ratio 1.66 [1.19–2.04]) and stroke (adjusted hazard ratio 1.48 [1.02–2.16]) than those without. In the meta-analysis, the pooled hazard ratio of ischemic heart disease and stroke were also increased in women with a history of preeclampsia (ischemic heart disease 1.65 [1.51–1.82]; stroke 1.78 [1.52–2.10]).

## Introduction

Pregnancy becomes a window period to check the risk of cardiovascular disease after pregnancy because experiencing adverse pregnancy outcomes during pregnancy may accentuate cardiovascular or metabolic risk factor^1–6^. Inflammation, endothelial dysfunction, and impaired hemodynamic adaptation are possible mechanisms by which adverse pregnancy outcomes progress as future cardiovascular risk factors^5^. Preeclampsia (PE) which is one of the representative adverse pregnancy outcomes, occurs in 2–5% of all pregnancies globally, and its incidence is increasing over the decades^7–15^. The mechanism by which preeclampsia occurs during pregnancy remains unresolved. It is known to be some women with preexisting conditions associated with endothelial cell activation or inflammation are more likely to develop preeclampsia^16^. In women with these vulnerabilities, additional detrimental remodeling and accelerated vascular aging after delivery can result in chronic hypertension, type 2 diabetes, hyperlipidemia, metabolic syndrome and obesity, all of which are risk factors for cardiovascular disease^17,18^. Some recent studies demonstrated that the long-term adverse effects of PE on women’s cardiovascular health^2,3,17–20^.

PE prevalence differs according to race and ethnicity. Many studies have reported an increased risk of PE in non-Hispanic Black women^21–23^. According to data on PE and eclampsia among inpatient deliveries in 2014 in the United States, Asian/Pacific Islanders were reported to have the lowest rate of PE compared to that in Black, White, and Hispanic women^24^. Racial and ethnic differences also affect the risk of cardiovascular disease (CVD). The rate of acute myocardial infarction was reported to be higher in black women aged 35–74 years than in Whites and Asian/Pacific Islander women^25^. The prevalence of coronary heart disease in the United States was lower in Asian women than in White, Hispanic, and Black women^26^. In contrast, Asian Americans women were more likely to have severe stroke than White women^27^.

Based on these racial/ethnic differences in both PE and CVD, it is presumed that the long-term cardiovascular risk of PE may also differ between races/ethnicities. However, most published studies were conducted in predominantly White women in Western countries. To date, only a few studies have been conducted in Asian populations, but the results have been conflicting. Cho et al. conducted study on 420,407 primigravid women using the database of the Korea National Health Insurance Service and National Health Screening Examination in South Korea and found an association between preeclampsia and ischemic heart disease after delivery (adjusted hazard ratio, aHR 1.66 [1.51–1.81])^28^. However, Wang et al. could not find this association (adjusted odds ratio 0.96 [0.60–1.53]) in their large-scale cohort study including 5807 primigravid women aged 15–40 using the universal insurance claims data in Taiwan^29^. In addition, long-term follow-up studies for several decades are lacking, as most previous cohort studies followed up women for less than 20 years after delivery for the occurrence of CVD.

In short, while there have been some studies targeting Asians, there hasn’t been a study on a large-scale population with a median follow-up duration of 22 years, focusing specifically on preeclampsia, like the one conducted in this research. In this study, we assessed the long-term risk of ischemic heart disease (IHD) and stroke in women with a history of PE using a large population-based cohort in South Korea. Furthermore, for a comprehensive analysis of the cardiovascular risk of PE in the Asian population, we also conducted a meta-analysis of published literature and the current study.

## Results

### HEXA cohort study in South Korea

**Figure 1:**
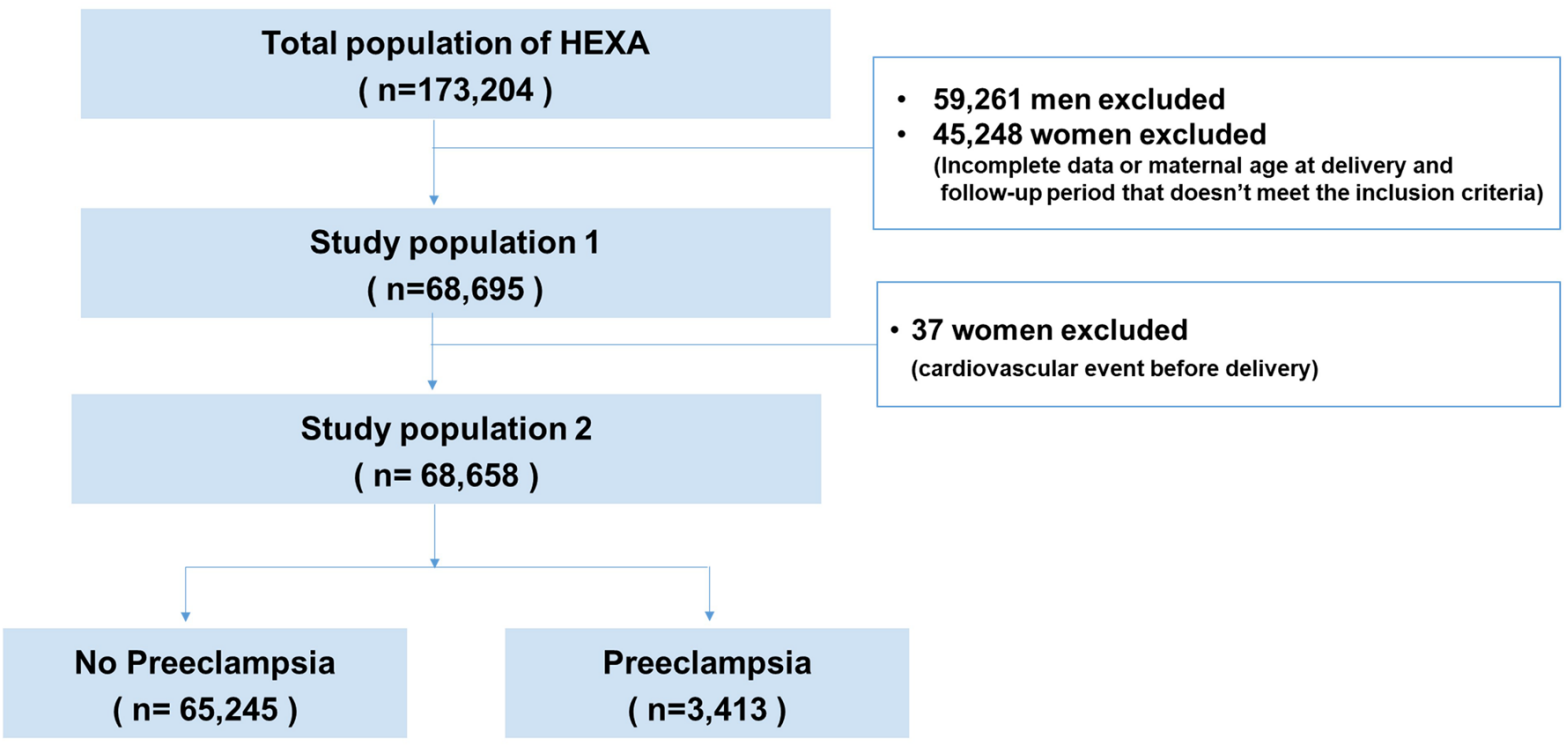
Flow diagram of the study population describing the inclusion and exclusion criteria of the HEXA study population.

This study included female participants who delivered between the ages of 18 and 45 years. Women with incomplete data and pre-existing IHD or stroke before delivery were excluded. In total, 68,658 women were included in the final analysis (Fig. 1). Among the study population, 3413 (5.23%) women had a history of PE during pregnancy. Table 1 shows the demographic and clinical characteristics of the study population. The ages of women at delivery with and without a history of PE were 30.4 and 30.1 years, respectively. Women with a history of PE had higher rates of GDM and delivery of low birth weight and macrosomia newborns than women without a history of PE (*p* < 0.001).

**Table 1:** Demographic and clinical characteristics of the HEXA study population.

	Control group (n = 65,245)	Preeclampsia group (n = 3413)	*p* value
Baseline characteristics			
Age at enrollment (years)	49.19 ± 6.01	49.13 ± 5.89	0.721
Duration between delivery and last follow up	20.52 ± 6.72	20.24 ± 6.80	0.025
Family history of stroke	8356 (7.44%)	409 (11.98%)	< 0.001
Family history of IHD	5087 (7.80%)	325 (9.52%)	< 0.001
Obstetric characteristics			
Age at delivery (years)	30.1 ± 3.9	30.4 ± 4.1	0.001
Parity	2.2 ± 0.8	2.2 ± 0.8	0.230
GDM during pregnancy	609 (0.93%)	244 (7.15%)	< 0.001
Delivery of LBW	2827 (4.33%)	483 (14.15%)	< 0.001
Delivery of macrosomia	4851 (7.44%)	409 (11.98%)	< 0.001
Prevalent comorbidity before delivery			
Diabetes	46 (0.07%)	4 (0.12%)	0.314
Hypertension	61 (0.09%)	20(0.59%)	< 0.001
Dyslipidemia	24 (0.04%)	2 (0.06%)	0.373

BMI, body mass index; GDM, gestational diabetes mellitus; IHD, ischemic heart diseases; LBW, low birth weight.

The median follow-up duration was 22 years (range: 16–26 years). Among the study population, 767 (1.12%) and 404 (0.59%) women experienced IHD and stroke after delivery, respectively. The incidence rates of both IHD and stroke after delivery per 1000 person-years were higher in women with a history of PE than in those without (IHD, 0.822 vs. 0.530, *p* < 0.001; stroke, 0.434 vs. 0.279, *p* < 0.001). In the Cox regression analysis, women with a history of PE had a 1.558 times higher risk of IHD and a 2.042 times higher risk of stroke than those without a history of PE (IHD adjusted hazard ratio [aHR] 1.558; 95% confidence interval [95% CI], 1.189–2.042; stroke aHR 1.483; 95% CI, 1.020–2.155) (Table 2).

**Table 2:** Incident diagnosis of cardiovascular outcomes in the HEXA study population.

Outcomes	Number of events		Crude incidence rate per 1000 person-years		Unadjusted HR (95% CI)	Model 1 HR (95% CI)	Model 2 HR (95% CI)	Model 3 HR (95% CI)
	Control	History of PE	Control	History of PE				
IHD	710/65,245 (1.09%)	57/3413 (1.67%)	0.530	0.822***	1.58***	1.59***	1.59***	1.56**
					(1.21–2.07)	(1.21–2.08)	(1.21–2.08)	(1.19–2.04)
Stroke	374/65,245 (0.57%)	30/3413 (0.88%)	0.279	0.434***	1.57*	1.57*	1.54*	1.48*
					(1.08–2.28)	(1.08–2.28)	(1.06–2.24)	(1.02–2.16)

Data are presented as proportion (%).

CI, confidence interval; CVD, cardiovascular diseases; HR, hazard ratio; IHD, ischemic heart diseases; PE, preeclampsia.

Significant codes: ‘***’ < 0.001; ‘**’ < 0.01; ‘*’ < 0.05.

Model 1, adjusted for age at delivery and parity using Cox proportional hazards regression analysis.

Model 2, adjusted for age at delivery, parity, and prevalent diseases (hypertension, diabetes, or dyslipidemia) using Cox proportional hazards regression analysis.

Model 3, adjusted for age at delivery, parity, prevalent diseases (hypertension, diabetes, or dyslipidemia), and family history (ischemic heart disease or stroke) using Cox proportional hazards regression analysis.

### Meta-analysis

**Figure 2:**
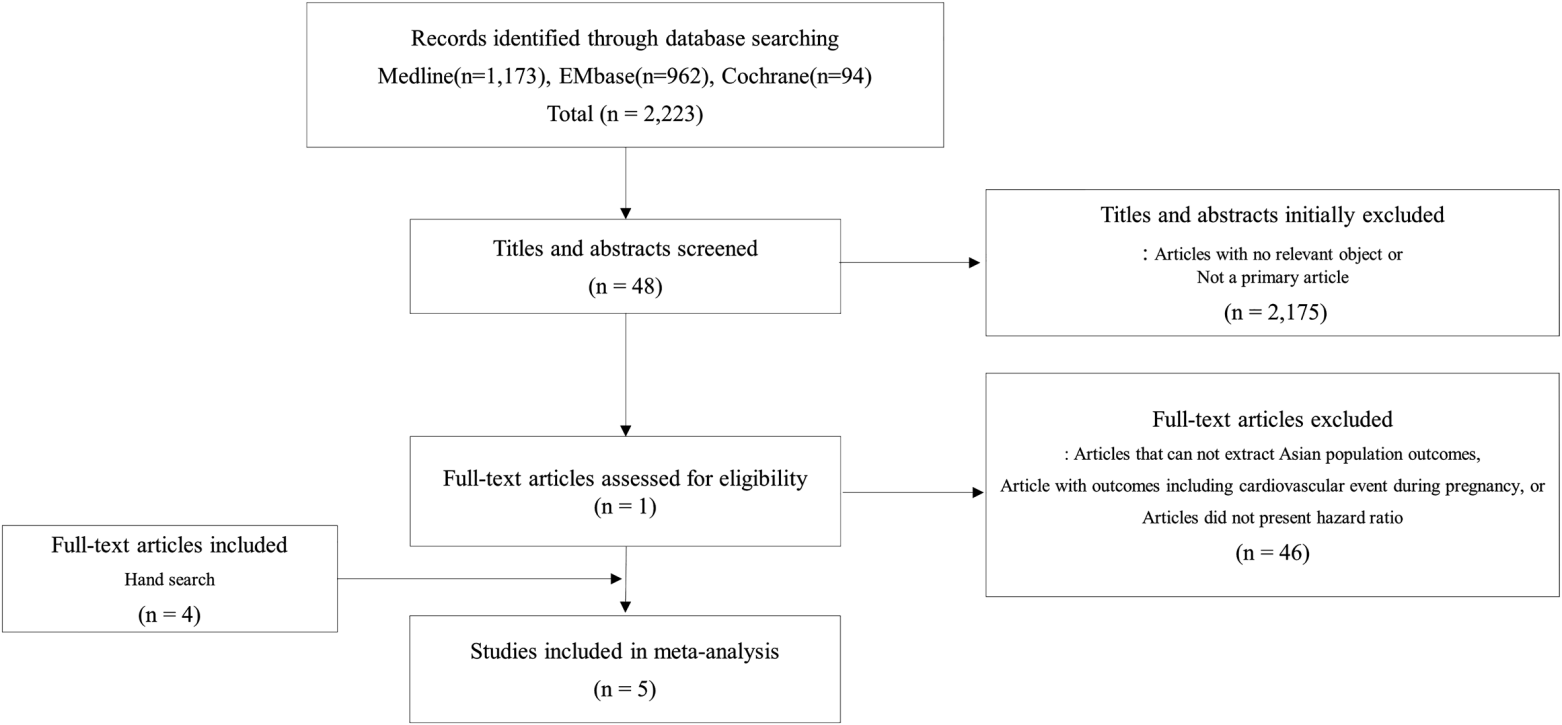
Flow diagram of the inclusion criteria of studies for meta-analysis.

To comprehensively assess the long-term cardiovascular risk of hypertensive disorders of pregnancy among Asian women, a meta-analysis, including the current study, was conducted. A total of 2223 studies were initially searched, and five studies, including three studies that were manually searched, met the inclusion criteria (Fig. 2)^28–32^. Among the five previous studies, three studies presented the cardiovascular risk of hypertensive disorders of pregnancy which was defined as gestational hypertension, PE, eclampsia, or superimposed PE^29,31,32^. Two studies presented the cardiovascular risk of PE (Table 3)^28,30^.

**Table 3:** Previous studies on cardiovascular risk of hypertensive disorders of pregnancy.

Study	Study design	Number of study population	Scope of hypertensive disorders of pregnancy	Follow-up duration (years)	aHR (95% CI)	Adjusted variables
Current study	Retrospective cohort	68,658	PE	22	IHD; 1.56 (1.19–2.04)	Age, parity, HTN, diabetes, dyslipidemia, and family history
				(IQR, 16–26)	Stroke; 1.48 (1.02–2.16)	
Wang^29^	Retrospective,	5807	Gestational hypertension, eclampsia, PE or superimposed PE	6.64 ± 1.57	Stroke; 2.04 (1.18–3.51)	Urbanization level, diabetes, hyperlipidemia, coronary artery disease, preterm delivery, placental abruption, lupus, and thrombophilia
	Case control					
Kuo^30^	Retrospective,	6324	PE	9.8	Stroke, 3.47 (1.46–8.23)	None
	Case control			(IQR, 5.1–12.7)		
Huang^32^	Retrospective,	167,480	gestational hypertension, eclampsia, PE or superimposed PE	13 (maximum)	Stroke; 2.13 (1.82–2.51)	Urbanization level, income, and season
	Case control					
Hung^31^	Retrospective,	68,085	gestational hypertension, eclampsia, PE or superimposed PE	17 (maximum)	Stroke; 1.71 (1.46–2.00)	Urbanization level, geographic region, income, season, age, mode of delivery, multiple gestation, multiple hypertensive disorders of pregnancy, hospital level, chronic HTN, GDM, anemia, antepartum hemorrhage, and postpartum hemorrhage
	Case control					
Cho^28^	Retrospective cohort	420,407	PE	10 (maximum)	IHD; 1.66 (1.51–1.82)	Age, BMI, systolic BP, diastolic BP, aspartate aminotransferase, alanine aminotransferase, fating glucose, total cholesterol, and smoking
					Stroke; 1.54 (1.39–1.71)	

aHR, adjusted hazard ratio; BMI, body mass index; BP, blood pressure; GDM, gestational diabetes mellitus; HTN, hypertension; IHD, ischemic heart disease; IQR, interquartile range; PE, preeclampsia.

Among the five previous studies, only one study^28^ assessed the risk of IHD, and all five studies^28–32^ assessed the risk of stroke among women with a history of hypertensive disorders of pregnancy.

**Figure 3:**
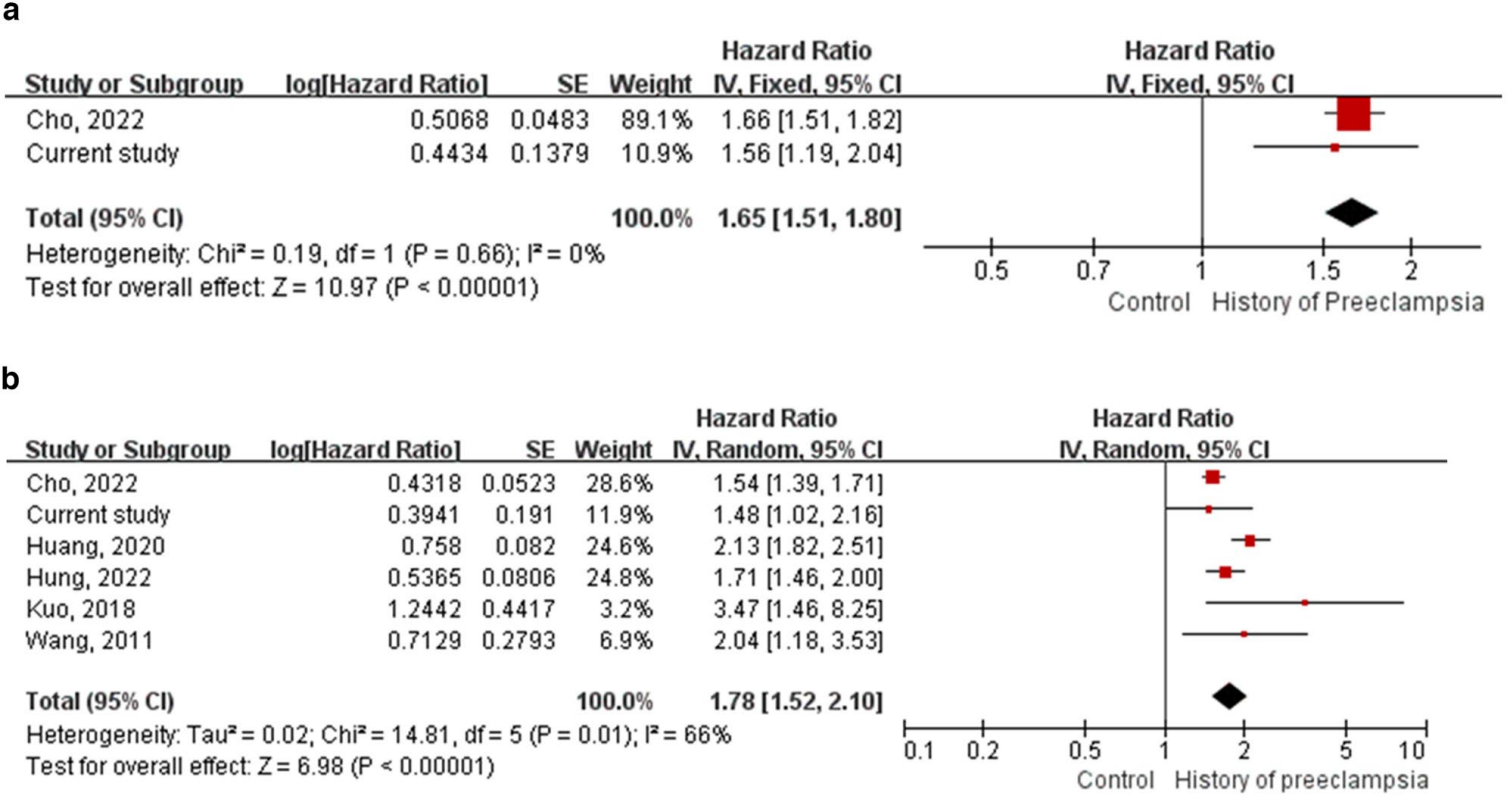
Hazard ratios of (**a**) ischemic heart disease and (**b**) stroke in women with a history of hypertensive disorders of pregnancy in the meta-analysis. (**a**) Hazard ratio of ischemic heart disease in women with a history of hypertensive disorders of pregnancy. (**b**) Risk ratio for stroke in women with a history of hypertensive disorders of pregnancy.

Including the current study, the total study population of the meta-analysis for long-term risk of hypertensive disorders of pregnancy on IHD was 489,065. Among them, 12,848 (2.63%) had a history of hypertensive disorders of pregnancy. The meta-analysis showed that women with hypertensive disorders of pregnancy were 1.65 times more likely to have IHD after delivery than those without (aHR, 1.65; 95% CI 1.51–1.80) (Fig. 3a).

The total study population of the meta-analysis for the long-term risk of hypertensive disorders of pregnancy on stroke was 736,912. Among them, 70,722 (9.60%) women had a history of hypertensive disorders of pregnancy. The results of the five previous studies were consistent with those of the current study, which showed an increased risk of stroke in women with a history of hypertensive disorders of pregnancy^28–32^. The meta-analysis also showed that women with a history of hypertensive disorders of pregnancy were 1.78 times more likely to have a stroke after delivery than those without (aHR, 1.78; 95% CI 1.52–2.10) (Fig. 3b).

## Discussion

The major findings of this study are as follows. (1) A history of PE increased the risk of both stroke and IHD in the HEXA cohort of South Korea, which followed women over 20 years after delivery. (2) In a meta-analysis of Asian populations, the risk of IHD and stroke after delivery was increased in women with a history of hypertensive disorders of pregnancy.

To the best of our knowledge, the current study evaluated the hazard ratio of incident CVD after delivery with the longest duration of follow-up in the Asian population. The median follow-up duration of the current study was 22 years, whereas that of previous studies was less than 20 years^28–32^. In this study, we found that the CVD risk of PE persisted for more than 20 years after delivery and remained beyond the middle age of Asian women. In contrast to a recent study in which the acute CVD risk of PE (during delivery hospitalization) was higher in Asian women than in White or Black women (HR of IHD, Asian 6.74; White 3.87; Black 2.55)^33^, the risk of long-term CVD of PE in Asian populations was similar to that of White women^34–37^.

The need to investigate the cardiovascular risk of PE in various races/ethnicities has been mentioned in previous studies^4,38,39^. Several studies have reported that black women with a history of PE are at a higher risk of CVD than other races; however, few studies have been conducted in the Asian population^17,40–43^. In addition, as it was mentioned before, results from previous studies in Asian women showed conflicting outcomes regarding CVD risk during pregnancy or after delivery. The analytic methods of previous studies suggesting CVD risk also varied, including odds ratio [OR], HR, or relative risk [RR]. Therefore, the evidence on long-term CVD risk of PE in Asian women is non-uniform and insufficient. To ascertain the long-term cardiovascular risk of PE in Asian women accurately, current study excluded studies which assessed CVD outcome during pregnancy or presented CVD risk other than HR from meta-analysis. We finally included five studies with a total of 731,105 women. The results of our meta-analysis confirmed that a history of PE increases the risk of IHD and stroke, even in Asian women.

Because adaptation to the physiologic changes of pregnancy is an advance warning of a woman’s risk of later CVD, the American Heart Association has updated its guidelines for CVD prevention in women, recommending consideration of a history of adverse pregnancy outcomes in the part of CVD risk assessment^44^. With large-scale prospective cohort (HEXA study) and meta-analysis in Asian women we were able to evaluate the risk of CVD after PE in a clear manner. Based on these results women should be monitored closely for the development of preeclampsia during pregnancy, and if it occurs, should expeditiously undergo comprehensive preventive screening and management for reducing CVD risk.

The strength of this study is that it has the longest follow-up period compared to other studies in the Asian population. In addition, we also presented the result of a meta-analysis on CVD risk of PE in the Asian population. Lastly, we adjusted for the risk factors of CVD that presented before delivery, thus reaffirming that the cardiovascular risk of PE remains after adjusting for well-known risk factors. This study has several limitations. First, we didn’t evaluate the impact of gestational hypertension, eclampsia, and superimposed PE while the meta-analysis assessed the impact of hypertensive disorders of pregnancy including PE, gestational hypertension, eclampsia or superimposed PE. This study focused on the impact of PE because PE is potentially more ominous and significant compared to gestational hypertension considering both disease severity and incidence. In addition, HEXA cohort only provided the data regarding PE. In addition, due to the lack of data, we were unable to stratify the subtypes of PE such as early-onset, late-onset or severe PE. Secondly, the main outcomes of the study, which are the occurrence of stroke and ischemic heart disease were identified based on the participants’ interview-based questionnaires, thereby leading to information bias. Another limitation was that the range of hypertensive disorder during pregnancy of each study included in the meta-analysis was heterogeneous. Some studies targeted the impact of hypertensive disorders of pregnancy which included gestational hypertension, PE, eclampsia, and superimposed PE^29,31,32^. While the current study, Kuo et al. and Cho et al. focused on preeclampsia^28,30^.

The current study presented the long-term cardiovascular risk of PE in Asian women. The long-term risk of both IHD and stroke in women with a history of preeclampsia lasted more than 20 years after delivery in Asian women. Therefore, surveillance of CVD in women with a history of PE should be maintained lifelong and not be limited to the postpartum period. Moreover, a large prospective cohort study on the long-term CVD of PE is needed to reaffirm the cardiovascular risk of PE in the Asian population.

## Methods

### Health examinees cohort study in South Korea

#### Study population

The Health Examinees (HEXA) study is a large-scale prospective cohort study that enrolled 167,169 residents of 14 urban and suburban areas in the Republic of Korea between January 2004 and December 2013^45^. For the longitudinal follow-up, participants were invited to attend the assessment center for the new occurrence of adverse outcomes between 2012 and 2016. The ethnicity of the participants was all Korean. The data regarding the participants’ race was not investigated. The study population was followed up until the last visit to the assessment center. The current study included female HEXA participants who delivered between the age of 18 and 45 years and reported at least one birth. Participants were followed for up to 30 years after delivery.

The risks of IHD and stroke after delivery were compared according to history of PE during pregnancy. In the current study, women with pre-existing IHD or stroke before delivery were excluded.

#### Data sources and definition of variables

At enrollment, the participants in the HEXA study provided informed consent and their information using an interview-based questionnaire regarding demographic factors, family history, and medical history including the diagnosis of disease and the age at first diagnosis, and obstetric history, including age at delivery and the diagnosis of obstetric complications such as gestational diabetes (GDM) and PE by a doctor^45^. In addition, the HEXA study followed participants until 2012–2016 regarding the new occurrence of medical diseases. Based on this interview-based questionnaires, the occurrence of IHD and stroke was identified. The occurrence of IHD was defined as the diagnosis of angina pectoris or myocardial infarction after the time of delivery. The occurrence of stroke was defined as the diagnosis of a stroke after the time of delivery. Prevalent comorbidities, including diabetes, hypertension, and dyslipidemia, were defined as those that occurred before the delivery.

#### Statistical analysis

Continuous variables are presented as mean and standard deviation. The chi-square test or Fisher’s exact test was used to analyze categorical variables, as appropriate. The Student’s t-test or Mann–Whitney U test was used for continuous variables, as appropriate. The incidence rates of IHD and stroke after delivery were calculated per 1000 person-years. The Cox proportional hazards model was used to evaluate hazard ratios and 95% confidence intervals for total IHD and stroke. The HRs for outcomes were adjusted for age at delivery, parity, prevalent hypertension/diabetes/dyslipidemia before delivery, family history of IHD, and family history of stroke. *p* < 0.05 was considered statistically significant. All statistical analyses were conducted using R Statistical Software (version 4.0.5; R Foundation for Statistical Computing, Vienna, Austria).

### Meta-analysis

#### Literature search

To evaluate the risk of IHD and stroke after hypertensive disease during pregnancy in an Asian population, we performed a meta-analysis combining data from the current study with data from the existing literature. To identify published studies, we searched with a trained librarian for all publications in English on the topic. We searched the terms for hypertensive disease during pregnancy (e.g., preeclampsia, gestational hypertension, or pregnancy-induced hypertension) and CVD (e.g., cardiovascular disease, stroke, myocardial ischemia, or coronary artery disease) using MEDLINE, EMBASE, and Cochrane databases for original articles. Additional studies were included in the manual search of the original articles (Supplementary Table 1; Supplementary Fig. 1).

#### Study selection

Original articles with two types of study design were included in the meta-analysis: cohort and case–control studies. Studies that assessed the risk of subsequent stroke or IHD in women with a history of hypertension during pregnancy were included. Studies that did not include Asian women in the study population or could not extract the results from the Asian population were excluded. Studies that included total CVD occurring during pregnancy as outcomes or those that did not present hazard ratios as outcomes were excluded. Three authors (E-SC, DYK, and SEC) screened the articles by title and abstract. The full articles were reviewed by the same reviewers, and the final study selection was performed by E-SC.

#### Data analysis

In the meta-analysis, the scope was expanded beyond PE and to analyze the impact of hypertensive disorders of pregnancy which includes gestational hypertension, PE, eclampsia or superimposed PE. We analyzed the data using RevMan version 5.4 (Nordic Cochrane Center) and used the inverse variance method to evaluate hazard ratios. In analyzing the risk of subsequent stroke after hypertensive disease during pregnancy, a random effects model was used, considering the heterogeneity of the study population of the included articles. However, in the analysis of subsequent IHD after hypertensive disease during pregnancy, a fixed effect analysis was performed because only two studies were available in this meta-analysis and variability between studies could not be estimated reliably^46,47^. We estimated the pooled hazard ratio using the adjusted risk from the selected original articles and the current stud

### Ethics approval and consent to participate

HEXA study followed a standard study protocol approved by the Ethics Committee of the Korean Health and Genomic Study of the Korean National Institute of Health and institutional review boards from all participating centers. The current study was approved by the Institutional Review Board of the Seoul National University Hospital approved this study. We confirm that all methods were performed in accordance with the relevant guidelines and regulation.

### Supplementary Information

Supplementary Information.

## Data Availability

The data that support the findings of this study are available from National Biobank of Korea, the Korea Disease Control and Prevention Agency, Republic of Korea, but restrictions apply to the availability of these data, which were used under license for the current study, and so are not publicly available. Data are however available from the corresponding authors upon reasonable request and with permission of National Biobank of Korea, the Korea Disease Control and Prevention Agency, Republic of Korea.

## References

[1] Cho GJ (2019). Is preeclampsia itself a risk factor for the development of metabolic syndrome after delivery?. Obstet. Gynecol. Sci..

[2] Markovitz AR (2019). Does pregnancy complication history improve cardiovascular disease risk prediction? Findings from the HUNT study in Norway. Eur. Heart J..

[3] Grandi SM (2019). Cardiovascular disease-related morbidity and mortality in women with a history of pregnancy complications. Circulation.

[4] Parikh NI (2021). Adverse pregnancy outcomes and cardiovascular disease risk: Unique opportunities for cardiovascular disease prevention in women: A scientific statement from the American Heart Association. Circulation.

[5] Hauspurg A, Ying W, Hubel CA, Michos ED, Ouyang P (2018). Adverse pregnancy outcomes and future maternal cardiovascular disease. Clin. Cardiol..

[6] Lee KH (2023). Long term renal outcome after hypertensive disease during pregnancy: A nationwide population-based study. Obstet. Gynecol. Sci..

[7] Ananth CV, Keyes KM, Wapner RJ (2013). Pre-eclampsia rates in the United States, 1980–2010: Age-period-cohort analysis. BMJ.

[8] Abalos E, Cuesta C, Grosso AL, Chou D, Say L (2013). Global and regional estimates of preeclampsia and eclampsia: A systematic review. Eur. J. Obstet. Gynecol. Reprod. Biol..

[9] Magee LA, Nicolaides KH, von Dadelszen P (2022). Preeclampsia. N. Engl. J. Med..

[10] Chappell LC, Cluver CA, Kingdom J, Tong S (2021). Pre-eclampsia. Lancet.

[11] Park Y (2018). Preeclampsia increases the incidence of postpartum cerebrovascular disease in Korean population. J. Korean Med. Sci..

[12] Centers for Disease Control and Prevention. *Data on selected pregnancy complications in the United States*, https://www.cdc.gov/reproductivehealth/maternalinfanthealth/pregnancy-complications-data.htm (2017).

[13] Cameron NA (2022). Trends in the incidence of new-onset hypertensive disorders of pregnancy among rural and urban areas in the United States, 2007 to 2019. J. Am. Heart Assoc..

[14] Yang Y (2021). Preeclampsia prevalence, risk factors, and pregnancy outcomes in Sweden and China. JAMA Netw. Open.

[15] Pare E (2014). Clinical risk factors for preeclampsia in the 21st century. Obstet. Gynecol..

[16] Cunningham FG (2022). Williams Obstetrics.

[17] Cirillo PM, Cohn BA (2015). Pregnancy complications and cardiovascular disease death: 50-Year follow-up of the Child Health and Development Studies pregnancy cohort. Circulation.

[18] Sattar N, Greer IA (2002). Pregnancy complications and maternal cardiovascular risk: Opportunities for intervention and screening?. BMJ.

[19] Canoy D (2016). Hypertension in pregnancy and risk of coronary heart disease and stroke: A prospective study in a large UK cohort. Int. J. Cardiol..

[20] Riise HKR (2019). Hypertensive pregnancy disorders increase the risk of maternal cardiovascular disease after adjustment for cardiovascular risk factors. Int. J. Cardiol..

[21] American College of Obstetricians and Gynecologists’ Committee on Practice Bulletin (2020). Gestational hypertension and preeclampsia: ACOG Practice Bulletin, Number 222. Obstet. Gynecol..

[22] Nakimuli A (2014). Pregnancy, parturition and preeclampsia in women of African ancestry. Am. J. Obstet. Gynecol..

[23] Breathett K, Muhlestein D, Foraker R, Gulati M (2014). Differences in preeclampsia rates between African American and Caucasian women: Trends from the National Hospital Discharge Survey. J. Womens Health.

[24] Fingar, K.R. *et al. Statistical Brief #222: Delivery Hospitalizations Involving Preeclampsia and Eclampsia, 2005–2014*, https://www.hcup-us.ahrq.gov/reports/statbriefs/sb222-Preeclampsia-Eclampsia-Delivery-Trends.pdf (2017). 28722848

[25] Chi GC (2020). Trends in acute myocardial infarction by race and ethnicity. J. Am. Heart Assoc..

[26] Virani SS (2021). Heart disease and stroke statistics-2021 update: A report from the American Heart Association. Circulation.

[27] Song S (2019). Comparison of clinical care and in-hospital outcomes of Asian American and white patients with acute Ischemic stroke. JAMA Neurol..

[28] Cho GJ (2022). Prior pregnancy complications and maternal cardiovascular disease in young Korean women within 10 years after pregnancy. BMC Pregnancy Childbirth.

[29] Wang IK (2011). Hypertensive disorders in pregnancy and preterm delivery and subsequent stroke in Asian women: A retrospective cohort study. Stroke.

[30] Kuo YL, Chan TF, Wu CY, Ker CR, Tu HP (2018). Preeclampsia-eclampsia and future cardiovascular risk among women in Taiwan. Taiwan J. Obstet. Gynecol..

[31] Hung SK (2022). Impact of hypertensive disorders of pregnancy on the risk of stroke stratified by subtypes and follow-up time. Stroke.

[32] Huang CC (2020). Association between hypertensive pregnancy disorders and future risk of stroke in Taiwan: A Nationwide population-based retrospective case-control study. BMC Pregnancy Childbirth.

[33] Minhas AS (2021). Racial disparities in cardiovascular complications with pregnancy-induced hypertension in the United States. Hypertension.

[34] Haug EB (2019). Association of conventional cardiovascular risk factors with cardiovascular disease after hypertensive disorders of pregnancy: Analysis of the Nord-Trondelag Health Study. JAMA Cardiol..

[35] Skjaerven R (2012). Cardiovascular mortality after pre-eclampsia in one child mothers: Prospective, population based cohort study. BMJ.

[36] Behrens I (2017). Risk of post-pregnancy hypertension in women with a history of hypertensive disorders of pregnancy: Nationwide cohort study. BMJ.

[37] Mongraw-Chaffin ML, Cirillo PM, Cohn BA (2010). Preeclampsia and cardiovascular disease death: Prospective evidence from the child health and development studies cohort. Hypertension.

[38] Burger RJ (2022). Hypertensive disorders of pregnancy and cardiovascular disease risk across races and ethnicities: A review. Front. Cardiovasc. Med..

[39] Khosla K (2021). Long-term cardiovascular disease risk in women after hypertensive disorders of pregnancy: Recent advances in hypertension. Hypertension.

[40] Shahul S (2015). Racial disparities in comorbidities, complications, and maternal and fetal outcomes in women with preeclampsia/eclampsia. Hypertens. Pregnancy.

[41] Weissgerber TL (2013). Hypertension in pregnancy is a risk factor for peripheral arterial disease decades after pregnancy. Atherosclerosis.

[42] Savitz DA, Danilack VA, Elston B, Lipkind HS (2014). Pregnancy-induced hypertension and diabetes and the risk of cardiovascular disease, stroke, and diabetes hospitalization in the year following delivery. Am. J. Epidemiol..

[43] Johnston A (2021). Use of race, ethnicity, and national origin in studies assessing cardiovascular risk in women with a history of hypertensive disorders of pregnancy. CJC Open.

[44] Mosca L (2011). Effectiveness-based guidelines for the prevention of cardiovascular disease in women–2011 update: A guideline from the American Heart Association. Circulation.

[45] Health Examinees Study Group (2015). The Health Examinees (HEXA) study: Rationale, study design and baseline characteristics. Asian Pac. J. Cancer Prev..

[46] Borenstein M, Hedges LV, Higgins JP, Rothstein HR (2010). A basic introduction to fixed-effect and random-effects models for meta-analysis. Res. Synth. Methods.

[47] Higgins JPT, Green S, Cochrane Collaboration (2008). Cochrane Handbook for Systematic Reviews of Interventions.

