# Supplementary material for: Long-term cardiovascular outcome in women with preeclampsia in Korea: a large population-based cohort study and meta-analysis
Source: Sci Rep. 2024 Mar 29;14:7480. doi: 10.1038/s41598-024-57858-6 (PMC10980767; doi:10.1038/s41598-024-57858-6)
Supplement: Supplementary file 1 — Supplementary Information. [file 41598_2024_57858_MOESM1_ESM.docx]

**Supplementary Figure 1.** **Meta-analysis search strategy and flow chart**

Records identified through database searching
Medline(1172), Embase (1540), Cochrane (128)
Total (n=2840)

Records after duplicates removed
(n =2223)

Records excluded
(n=2175)

Review, Meta-analysis, Case report, Editorial, Conference abstract, Expert opinion, Comment, Letter, Study protocol, Studies with irrelevant objectives, Studies which do not analyze risk of ischemic heart disease or stroke, Studies which do not include Asian population

Full-text articles assessed for eligibility
(n=2)

Full-text articles excluded
(n=46)

Articles which cannot extract Asian population outcomes (n=41)

Article with outcomes including cardiovascular event during pregnancy (n=2)

Articles which did not present hazard ratio (n=2)

Articles which did not present the risk of stroke and ischemic heart disease respectively (n=1)

Studies included in meta-analysis
(n=5)

Titles and abstract screened
(n=48)

Full-text article included

Hand search

(n=3)

**Supplementary Table 1. Meta-analysis search strategy and flow chart**

| **Databases searched** | **Search** | **Search terms** | **Years of search** | **Number of searches** |
| --- | --- | --- | --- | --- |
| ovid Medline | 1 | exp Hypertension, Pregnancy-Induced/ | From the time each databases provided - July 2022 | 40,927 |
|  | 2 | "Gestational Hypertension*".ab,ti. |  | 3,533 |
|  | 3 | (Hypertens* adj5 pregnanc*).ab,ti. |  | 14,657 |
|  | 4 | "Pre-Eclampsia*".ab,ti. |  | 11,637 |
|  | 5 | "Preeclampsia*".ab,ti. |  | 25,060 |
|  | 6 | 1 or 2 or 3 or 4 or 5 |  | 60,294 |
|  | 7 | exp Myocardial Ischemia/ |  | 460,577 |
|  | 8 | ((Cardiovascular or Heart or Vascular) adj5 (Disease* or failure* or Stroke* or Attack*)).ab,ti. |  | 623,223 |
|  | 9 | "Myocardial Ischemia*".ab,ti. |  | 28,706 |
|  | 10 | "myocardial infarc*".ab,ti. |  | 212,617 |
|  | 11 | "coronary artery disease*".ab,ti. |  | 93,401 |
|  | 12 | "Coronary Atheroscleros*".ab,ti. |  | 8,400 |
|  | 13 | exp Brain Infarction/ |  | 41,247 |
|  | 14 | (Brain adj3 Infarct*).ab,ti. |  | 7,043 |
|  | 15 | exp Myocardial Infarction/ |  | 188,703 |
|  | 16 | exp Brain Ischemia/ |  | 120,410 |
|  | 17 | (Ischemi* adj5 (Brain* or Encephalopath* or Cerebral* or Hypoxi* or Anoxi*)).ab,ti. |  | 64,646 |
|  | 18 | "Metabolic Cardiovascular Syndrome*".ab,ti. |  | 53 |
|  | 19 | "Cardiometabolic Syndrome*".ab,ti. |  | 373 |
|  | 20 | 7 or 8 or 9 or 10 or 11 or 12 or 13 or 14 or 15 or 16 or 17 or 18 or 19 |  | 1,175,353 |
|  | 21 | exp Time/ |  | 1,410,745 |
|  | 22 | long term.ab,ti. |  | 916,243 |
|  | 23 | longterm.ab,ti. |  | 6,643 |
|  | 24 | exp Cohort Studies/ |  | 2,371,259 |
|  | 25 | "Cohort Stud*".ab,ti. |  | 278,609 |
|  | 26 | "Cohort Analys*".ab,ti. |  | 10,210 |
|  | 27 | 21 or 22 or 23 or 24 or 25 or 26 |  | 4,230,010 |
|  | 28 | 6 and 20 and 27 |  | 1,172 |
| Embase | #28 | #6 AND #20 AND #27 | From the time each databases provided - July 2022 | 1,540 |
|  | #27 | #21 OR #22 OR #23 OR #24 OR #25 OR #26 |  | 2,870,671 |
|  | #26 | 'cohort analys*':ab,ti |  | 16,368 |
|  | #25 | 'cohort stud*':ab,ti |  | 401,845 |
|  | #24 | 'cohort analysis'/exp |  | 863,899 |
|  | #23 | longterm:ab,ti |  | 1,120,213 |
|  | #22 | 'long term':ab,ti |  | 1,267,349 |
|  | #21 | 'time'/exp |  | 784,598 |
|  | #20 | #7 OR #8 OR #9 OR #10 OR #11 OR #12 OR #13 OR #14 OR #15 OR #16 OR #17 OR #18 OR #19 |  | 1,689,812 |
|  | #19 | 'cardiometabolic syndrome*':ab,ti |  | 521 |
|  | #18 | 'metabolic cardiovascular syndrome*':ab,ti |  | 58 |
|  | #17 | (ischemi* NEAR/5 (brain* OR encephalopath* OR cerebral* OR hypoxi* OR anoxi*)):ab,ti |  | 88,842 |
|  | #16 | 'brain ischemia'/exp |  | 208,759 |
|  | #15 | 'heart infarction'/exp |  | 439,523 |
|  | #14 | (brain NEAR/3 infarct*):ab,ti |  | 10,166 |
|  | #13 | 'brain infarction'/exp |  | 84,469 |
|  | #12 | 'coronary atheroscleros*':ab,ti |  | 12,349 |
|  | #11 | 'coronary artery disease*':ab,ti |  | 149,255 |
|  | #10 | 'myocardial infarc*':ab,ti |  | 315,054 |
|  | #9 | 'myocardial ischemia*':ab,ti |  | 39,167 |
|  | #8 | ((cardiovascular OR heart OR vascular) NEAR/5 (disease* OR failure* OR stroke* OR attack*)):ab,ti |  | 941,636 |
|  | #7 | 'heart muscle ischemia'/exp |  | 98,682 |
|  | #6 | #1 OR #2 OR #3 OR #4 OR #5 |  | 78,450 |
|  | #5 | 'preeclampsia*':ab,ti |  | 54,857 |
|  | #4 | 'pre-eclampsia*':ab,ti |  | 17,075 |
|  | #3 | (hypertens* NEAR/5 pregnanc*):ab,ti |  | 22,120 |
|  | #2 | 'gestational hypertension*':ab,ti |  | 5,731 |
|  | #1 | 'maternal hypertension'/exp |  | 27,848 |
| Cochrane library | #1 | MeSH descriptor: [Hypertension, Pregnancy-Induced] explode all trees | From the time each databases provided - July 2022 | 1,248 |
|  | #2 | (Gestational Hypertension*):ti,ab,kw |  | 1,689 |
|  | #3 | (Hypertens* near/5 pregnanc*):ti,ab,kw |  | 1,984 |
|  | #4 | (Pre-Eclampsia*):ti,ab,kw |  | 2,070 |
|  | #5 | (Preeclampsia*):ti,ab,kw |  | 3,823 |
|  | #6 | {OR #1-#5} |  | 5,529 |
|  | #7 | MeSH descriptor: [Myocardial Ischemia] explode all trees |  | 30,048 |
|  | #8 | (((Cardiovascular or Heart or Vascular) near/5 (Disease* or failure* or Stroke* or Attack*))):ti,ab,kw |  | 95,852 |
|  | #9 | (Myocardial Ischemia*):ti,ab,kw |  | 9,977 |
|  | #10 | (myocardial infarc*):ti,ab,kw |  | 33,638 |
|  | #11 | (coronary artery disease*):ti,ab,kw |  | 25,290 |
|  | #12 | (Coronary Atheroscleros*):ti,ab,kw |  | 3,934 |
|  | #13 | MeSH descriptor: [Brain Infarction] explode all trees |  | 1,429 |
|  | #14 | ((Brain near/3 Infarct*)):ti,ab,kw |  | 1,787 |
|  | #15 | MeSH descriptor: [Myocardial Infarction] explode all trees |  | 11,777 |
|  | #16 | MeSH descriptor: [Brain Ischemia] explode all trees |  | 4,024 |
|  | #17 | ((Ischemi* near/5 (Brain* or Encephalopath* or Cerebral* or Hypoxi* or Anoxi*))):ti,ab,kw |  | 8,483 |
|  | #18 | (Metabolic Cardiovascular Syndrome*):ti,ab,kw |  | 3,006 |
|  | #19 | (Cardiometabolic Syndrome*):ti,ab,kw |  | 849 |
|  | #20 | {OR #7-#19} |  | 143,330 |
|  | #21 | MeSH descriptor: [Time] explode all trees |  | 72,221 |
|  | #22 | (long term):ti,ab,kw |  | 109,152 |
|  | #23 | (longterm):ti,ab,kw |  | 93,000 |
|  | #24 | MeSH descriptor: [Cohort Studies] explode all trees |  | 160,327 |
|  | #25 | (Cohort Stud*):ti,ab,kw |  | 59,695 |
|  | #26 | (Cohort Analys*):ti,ab,kw |  | 40,985 |
|  | #27 | {OR #21-#26} |  | 340,585 |
|  | #28 | #6 and #20 and #27 |  | 128 |
